# Incidence and severity prediction score of COVID-19 in people living with HIV (SCOVHIV): experience from the first and second waves of the pandemic in Indonesia

**DOI:** 10.1186/s12981-022-00472-1

**Published:** 2022-10-03

**Authors:** Evy Yunihastuti, Teguh Harjono Karjadi, Alvina Widhani, Haridana Indah Setiawati Mahdi, Salma Sundari, Aljira Fitya Hapsari, Sukamto Koesnoe, Samsuridjal Djauzi

**Affiliations:** 1 Division of Allergy and Clinical Immunology, Department of Internal Medicine, Faculty of Medicine Universitas Indonesia, Cipto Mangunkusumo Hospital, Jl. Diponegoro no. 71, Jakarta, 10430 Indonesia; 2 HIV Integrated Unit, Cipto Mangunkusumo Hospital, Jakarta, Indonesia; 3 Sentra Medika Hospital, Depok, West Java Indonesia; 4 Department of Internal Medicine, Universitas Indonesia Hospital, Depok, West Java Indonesia; 5 Department of Non-Oncology Internal Medicine, Dharmais National Cancer Hospital, Jakarta, Indonesia

**Keywords:** COVID-19, HIV, Incidence, Indonesia, Severity prediction score

## Abstract

**Background:**

People living with HIV (PLHIV) have higher risk of COVID-19 infection and mortality due to COVID-19. Health professionals should be able to assess PLHIV who are more likely to develop severe COVID-19 and provide appropriate medical treatment. This study aimed to assess clinical factors associated with COVID-19 severity and developed a scoring system to predict severe COVID-19 infection among PLHIV.

**Methods:**

This retrospective cohort study evaluated PLHIV at four hospitals diagnosed with COVID-19 during the first and second wave COVID-19 pandemic in Indonesia. The independent risk factors related to the severity of COVID-19 were identified with multivariate logistic regression.

**Results:**

342 PLHIV were diagnosed with COVID-19, including 23 with severe-critical diseases. The cumulative incidence up to December 2021 was 0.083 (95% CI 0.074–0.092). Twenty-three patients developed severe-critical COVID-19, and the mortality rate was 3.2% (95% CI 1.61%–5.76%). Having any comorbidity, CD4 count of < 200 cells/mm^3^, not being on ART, and active opportunistic infection were independent risk factors for developing severe COVID-19. SCOVHIV score was formulated to predict severity, with 1 point for each item. A minimum score of 3 indicated a 58.4% probability of progressing to severe COVID-19. This scoring system had a good discrimination ability with the area under the curve (AUC) of 0.856 (95% CI 0.775–0.936).

**Conclusion:**

SCOVHIV score, a four-point scoring system, had good accuracy in predicting COVID-19 severity in PLHIV.

## Introduction

The coronavirus-19 (COVID-19) pandemic has impacted many people's health and economic status, especially people living with HIV (PLHIV). A meta-analysis has shown that PLHIV had a higher risk of contracting COVID-19 compared to HIV negative individuals [1]. However, health professionals are still investigating how COVID-19 affects PLHIV. HIV has been associated with an increased risk of death and severe outcomes in some meta-analyses [2–5]. Understanding the factors related to severe COVID-19 infection, including HIV-specific factors, is important as it will enable doctors to properly assess and prioritize COVID-19 treatment efforts. Furthermore, PLHIV have concerns about the COVID-19 severity risk related to their condition and would benefit from specific information on severity factors [6, 7].

Previous studies on factors related to COVID-19 severity among PLHIV showed conflicting results, except for comorbidities, as seen in the non-HIV population [8–10]. Some studies have shown that older PLHIV with lower CD4 count have a higher risk of severe outcomes. Other studies have shown opposing results, indicating that it is unclear whether CD4 count and viral load are indeed factors related to COVID-19 outcomes among PLHIV [11]. Differences in demographic and epidemiologic characteristics between comparison groups must also be taken into consideration. This study aimed to describe the cumulative incidence of SARS-CoV-2 in the first and second waves of the pandemic, as well as COVID-19 outcomes among people living with HIV in Indonesia. The 1st wave started in February 2021 and 2nd wave started in June 2021. Finally, this study attempted to assess clinical factors associated with COVID-19 severity and develop a predictive scoring system for severe COVID-19 infection.

## Methods

### Study population

This retrospective cohort study evaluated all adult PLHIV engaged in care at four participating HIV treatment centers: Cipto Mangunkusumo National Hospital (Jakarta), Dharmais National Cancer Hospital (Jakarta), Sentra Medika Hospital (West Java), and Universitas Indonesia Hospital (West Java). During the pandemic, COVID-19 diagnoses have been routinely collected at all sites. All cases of PLHIV who had documented SARS-CoV-2 infections as of December 2021 were included in the COVID-19 outcomes evaluation. SARS-CoV-2 infection was defined as a positive SARS-CoV-2 polymerase chain reaction (PCR) or SARS-CoV-2 antigen result.

### Data collection

A standardized form containing demographic and clinical information was used to extract data from each hospital’s medical records. Clinical parameters collected included body mass index (BMI), HIV-specific data (opportunistic infection, recent absolute CD4 count and viral load prior to COVID-19 infection, and antiretroviral used), as well as COVID-19 data (severity, hospitalization, and mortality). The BMI category for determining obesity was defined as a body mass index of 30 or higher [12]. Comorbidities evaluated included diabetes mellitus, hypertension, chronic kidney disease, cardiovascular disease, and obesity. Predetermined clinical outcomes of interest were COVID-19 severity and mortality. Severity was divided into several categories as follows: asymptomatic, mild (no pneumonia), moderate (pneumonia without oxygen supplementation), severe (pneumonia with oxygen supplementation), critical (requiring intensive care management or resulting in death by respiratory failure, septic shock, or multiple organ dysfunction or failure) [13, 14]. A severe COVID-19 outcome was a combination of severe and critical categories. Mortality was defined as all causes of mortality during COVID-19 infection.

### Statistical analysis

The cumulative incidence from the first clinically detected SARS-CoV-2 infection among PLHIV by key characteristics was calculated from March 1, 2020, until December 31, 2020. Frequencies and percentages were used for categorical variables, and continuous variables were expressed as a mean with SD or median and IQR on the imputed datasets. Missing values were imputed for education (3.5%), working status (0.9%) and recent CD4 count (5.5%). Bivariate analysis was performed to compare factors associated with severe COVID-19 outcomes. Multivariable backward logistic regression identified independent correlations with outcomes and calculated adjusted risk ratios (aRR) with a 95% confidence interval (CI). Differences in p-values of less than 0.05 were considered significant.

Factors that were found to be significant to COVID-19 severity outcomes were put into a predictive scoring system. Scores for each factor were calculated using a stepwise method as follows: (1) dividing risk factor’s coefficient B by its standard error (coefficient B/SE = x); (2) choosing the lowest x value as a reference for the next step; and (3) dividing each x value by the reference value. Calibration of the scoring, using Hosmer-Lemeshow and describing AUCS, was then performed to evaluate the scoring system. For internal validity, calibrations were validated using the bootstrap method. The analysis used STATA/IC for Windows version 14.2 (STATACorp LP).

## Results

### Incidence and characteristics of PLHIV with COVID-19

**Fig. 1:**
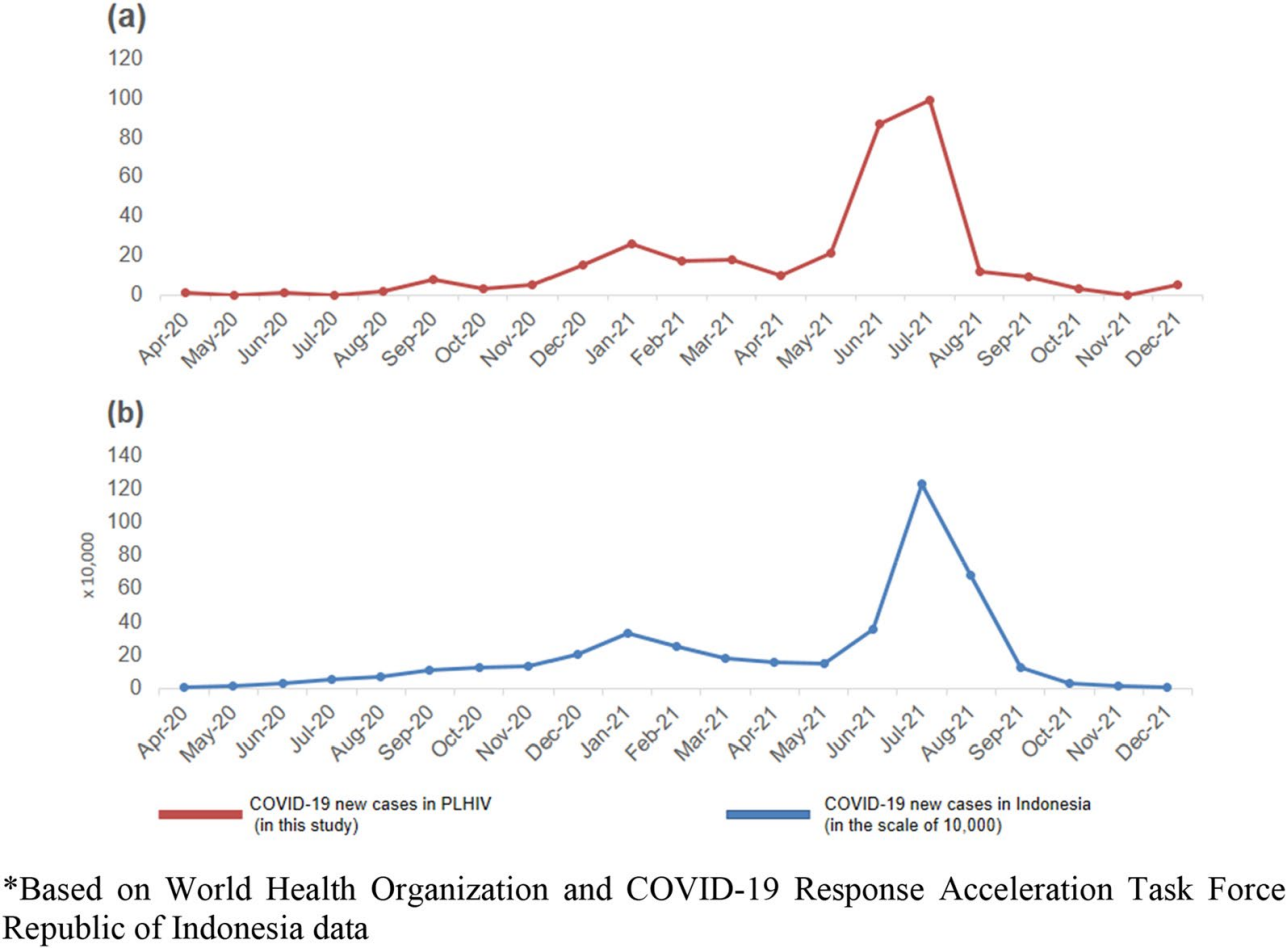
Comparison of COVID-19 new cases in PLHIV (in this study) **a** and in the Indonesian population* **b** from April 2020 to December 2021. Figure 1b is in the scale of 10,000.

During the study period, 342 PLHIV were confirmed to have SARS-CoV-2 infection from a total of 4134 PLHIV. In the same period, 4.6 million Indonesian people were diagnosed with COVID-19. The trend of COVID-19 infection in PLHIV almost mirrored the trend of COVID-19 cases in Indonesian population during the first wave and second wave as presented in Fig. 1. Most of the COVID-19 cases in Indonesia occurred during the second wave of the pandemic. The cumulative incidence of COVID-19 at the end of 2021 was 0.083 (95% CI 0.074–0.092) for adult PLHIV.

Most of the PLHIV with a confirmed SARS-CoV-2 infection were male (*n* = 259, 75.7%), acquired HIV via heterosexual transmission (*n* = 158, 46.2%), and were actively working (*n* = 266, 77.8%). Thirty-four PLHIV (9.9%) still had active opportunistic infections and twenty-seven (7.9%) had active tuberculosis infection. Sixty-four of the PLHIV (18.7%) had one or more of the following comorbidities: hypertension (*n* = 29, 8.5%), obesity (*n* = 27, 7.9%), diabetes (*n* = 13, 3.8%), chronic kidney disease (*n* = 7, 2%), or cardiovascular disease (*n* = 5, 1.5%).

Of the confirmed COVID-19 cases among the PLHIV in this study, 295 cases (86.3%) were asymptomatic or categorized as mild, 24 (7%) were moderate cases, 13 (3.8%) were severe cases, and 10 (2.9%) were critical cases, as can be seen in Table 1. Fourteen percent of the patients required hospitalization (48 cases) and eleven patients died during the course of their COVID-19 infections (mortality rate 3.2%, 95% CI 1.61–5.76%). Most of the COVID-19 patients self-quarantined (*n* = 287, 83.9%) while seven patients (2.1%) quarantined at the isolation center. Fifteen patients (4.4%) were unaware of their HIV status before becoming infected with COVID-19, and two patients were newly diagnosed HIV and not on antiretrovirals yet.

**Table 1:** Demographic and clinical characteristics of COVID-19 patients

	Total (*n* = 342)
Demographic Characteristics	
Male gender [*n* (%)]	259 (75.7)
Age in years, median (IQR)	39 (33 − 44)
Education [*n* (%)]	
Low	6 (1.8)
Middle	142 (41.5)
High	194 (56.7)
Working status [*n* (%)]	
Actively working	266 (77.8)
Not working	76 (22.2)
Marital status [*n* (%)]	
Married	166 (48.5)
Widowed	28 (8.2)
Not married	148 (43.3)
HIV transmission risk [*n* (%)]	
Heterosexual	158 (46.2)
Homosexual	93 (27.2)
IVDU	72 (21.1)
Unknown	19 (5.6)
Years of HIV diagnosis [*n* (%)]	
Newly diagnosed	15 (4.4)
≤ 1 year	36 (10.5)
2 − 5 years	85 (24.9)
6 − 10 years	104 (30.4)
> 10 years	102 (29.8)
Clinical characteristics	
Using ART before COVID-19 [*n* (%)]	320 (93.6)
Recent CD4 [*n* (%)]	
< 200 cells/mm^3^	48 (14)
≥ 200 cells/mm^3^	294 (86)
HIV viral load [*n* (%)]	
Virally suppressed	17 (5)
Not virally suppressed	192 (56.1)
No data	133 (38.9)
ART regimen (n = 320)	
Anchor drugs [*n* (%)]	
Nevirapine	85 (26.6)
Efavirenz	168 (52.5)
Lopinavir/ritonavir	45 (14.1)
Dolutegravir	22 (6.9)
Backbone drugs [*n* (%)]	
Tenofovir	203 (63.4)
Zidovudine	117 (36.6)
Opportunistic infection* [*n* (%)]	34 (9.9)
Tuberculosis [*n* (%)]	27 (7.9)
Obesity (BMI ≥ 30) [*n* (%)]	27 (7.9)
Diabetes [*n* (%)]	13 (3.8)
Hypertension [*n* (%)]	29 (8.5)
Chronic kidney disease [*n* (%)]	7 (2)
Cardiovascular disease [*n* (%)]	5 (1.5)
Any comorbidity [*n* (%)]	64 (18.7)

*IQR* interquartile range, *IVDU* intravenous drug user, *ART* antiretroviral therapy, *BMI* body mass index

^*^Pulmonary Tuberculosis, Extrapulmonary Tuberculosis (EPTB), Cytomegalovirus, Toxoplasmic Encephalitis, Esophageal Candidiasis, Cryptococcosis, Histoplasmosis

### Risk factors for severe COVID-19

Among the PLHIV with COVID-19 infection, bivariate analysis as seen in Table 2, showed that severe COVID-19 infection was associated with not using ART, a recent absolute CD4 count of less than 200 cells/mm^3^, having an active opportunistic infection, and having any comorbidity. In the adjusted model, not using ART was associated with an increased rate of severe COVID-19 infection (aRR = 3.15, 95% CI 1.616.19), as was a recent absolute CD4 count of less than 200 cells/mm^3^ (aRR = 2.15, 95% CI 1.12–4.14) and having an opportunistic infection or any comorbidity (aRR = 3.21, 95% CI 1.64–6.27 and aRR = 3.65, 96% CI 1.83–7.28).

**Table 2:** Bivariate analysis of factors related to severe COVID-19 infection

Variable	Severe-critical	Asymptomatic to moderate	RR (95%CI)	*P*
Gender [*n* (%)]				
Male	17 (73.9)	242 (75.9)	0.91 (0.37 − 2.23)	1.000
Female	6 (26.1)	77 (24.1)		
Age [*n* (%)]				
≥ 50 years	4 (17.4)	38 (11.9)	1.50 (0.54 − 4.21)	0.505
< 50 years	19 (82.6)	281 (88.1)		
BMI [*n* (%)]				
Obese (≥ 30)	4 (17.4)	23 (7.2)	2.46 (0.90 − 6.70)	0.096
Non-obese (< 30)	19 (82.6)	296 (92.8)		
Antiretroviral status [*n* (%)]				
Not-using ART	7 (30.4)	15 (4.7)	6.36 (2.93 − 13.83)	< 0.001
On ART	16 (69.6)	304 (95.3)		
Recent absolute CD4 [*n* (%)]				
< 200	11 (47.8)	37 (11.6)	5.62 (2.63 − 12.00)	< 0.001
≥ 200	12 (52.2)	282 (88.4)		
Opportunistic infection [*n* (%)]				
Active OI	9 (39.1)	25 (7.8)	5.82 (2.73 − 12.44)	< 0.001
No active OI	14 (60.9)	294 (92.2)		
Any comorbidity* [*n* (%)]				
Yes	11 (47.8)	53 (16.6)	3.98 (1.84 − 8.61)	0.001
No	12 (52.2)	266 (83.4)		

*BMI* body mass index, *ART* antiretroviral therapy, *OI* opportunistic infection

^*^diabetes, hypertension, chronic kidney disease, cardiovascular disease

### SCOVHIV: a scoring model for the prediction of severe COVID-19 among PLHIV

**Fig. 2:**
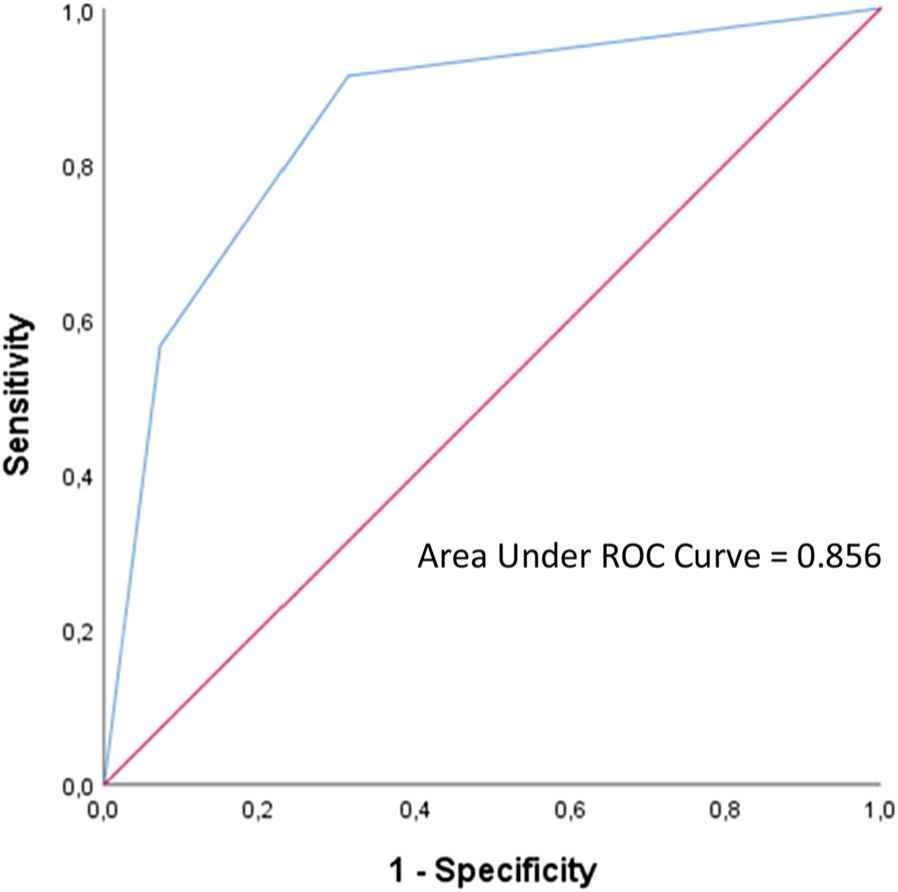
ROC *(Receiver Operating Characteristics)* for a 4-point scoring system as a predictor of COVID-19 severity among PLHIV

This study established a 4-point scoring system based on the adjusted model, and the results were put into bootstrapping logistic regression. The 4-point scoring system as seen in Table 3, covers the four risk factors included in the final multivariate model, which are ART status, recent absolute CD4 count, opportunistic infection, and any comorbidity. This scoring system had good discrimination ability with area under the curve (AUC) of 0.856 (95% CI 0.775–0.936; *p* < 0.01) and good calibration (Hosmer–Lemeshow test, 0.201) as seen in Fig. 2. Internal validity was also good (Hosmer–Lemeshow test: 0.201, p < 0.001). A minimum total score of 3 means that the probability of getting severe COVID-19 infection in PLHIV is 58.4%, while total scores of 2 or 1 mean the probability of getting severe COVID-19 infection is 25% and 7.3%, respectively.

**Table 3:** Derivation of 4-point scoring system to predict severity outcomes of COVID-19 infection from stepwise multivariate analysis (*n* = 342)

Variable	Coef. B	SE	*P value*	aRR (95% CI)	Score
Any comorbidity*	1.466	0.495	< 0.001	3.65 (1.83 − 7.28)	1
Recent absolute CD4 < 200 cells/mm^3^	1.585	0.515	0.022	2.15 (1.12 − 4.14)	1
Not using ART	1.483	0.596	0.001	3.15 (1.61 − 6.19)	1
Opportunistic infection	1.239	0.542	0.001	3.21 (1.64 − 6.27)	1

*ART* antiretroviral therapy

^*^diabetes, hypertension, obesity, chronic kidney disease, cardiovascular disease

## Discussion

COVID-19 incidence in this study was 0.083 (95% CI 0.074–0.092), which is higher than a study in Madrid [(0.067 (95% CI 0.057–0.079) [14]. The pooled incidence proportion in 7 other studies was 0.009 (95% CI 0.006–0.011), but this meta-analysis only represented studies in 2020 [15]. Most of the COVID-19 cases observed in this study had no symptoms or mild symptoms (86.3%). Rial-Crestelo et al. also observed a high proportion of patients with asymptomatic or mild disease in Spain (75.3% of 158 patients) [14], while a study in China showed 60% of 2464 PLHIV with COVID-19 infection being asymptomatic [16]. The proportion of severe and critical cases was smaller in our study (6.7%), which showed different patterns, although with limited numbers of COVID-19 cases in PLHIV. Mirzaei et al., which summarized 212 available data from 25 case reports and small studies, described the proportion of severe-critical case as 33.5% of all PLHIV with COVID-19 infection [5].

This study observed a lower mortality rate (3.2%) compared to previous reports. Ssentongo et al. showed pooled mortality rate for studies in 2020 was 12.65% (95% CI 6.81–22.31%) [1], while Liang et al. calculated a pooled mortality rate of 14 studies up to March 2021 was 8.814% [17]. We believe that more participants and longer observation time can better describe the proportion. However, these findings must be interpreted cautiously since a recent meta-analysis still found that PLHIV have a higher risk of death compared to non-HIV COVID-19 patients (HR = 1.76, 95% CI 1.31–2.35) [2].

Moreover, COVID-19 vaccination in PLHIV might change morbidity and mortality rates in the future. This study reported COVID-19 cases from March 2020 to December 2021 while the Indonesian government just started COVID-19 vaccination program to general population in June 2021. Therefore, we believe that not many COVID-19 patients in this study had been vaccinated though the data was not available.

The hospitalization rate of PLHIV with COVID-19 infection was found to be 14%, which is still within the range of other studies (13.8–58%) [9, 11, 14, 15, 18]. This low proportion of hospitalization does not necessarily mean that the number of patients who needed hospitalization was low. As shown in Fig. 1 [19, 20], more than half of the COVID-19 cases occurred during the second wave of the pandemic (June to August 2021) when the delta variant spread, although genomic sequencing was not routinely performed. Some patients may have had difficulties finding hospital care due to the limited capacities of health facilities during the increasing demand for hospitals experienced during waves [21].

Fifteen patients (4.4%) were unaware of their HIV diagnosis before contracting COVID-19. In the newly-diagnosed HIV patients, there were several reports of co-infection of COVID-19 and *Pneumocystic jirovecii* pneumonia [22, 23]. Some opportunistic pulmonary infections have similar clinical and radiological symptoms to COVID-19, including cytomegalovirus and *Pneumocystic jirovecii* pneumonia. Thus, other causes of respiratory infection than SARS-CoV-2 must still be considered even during this massive pandemic.

This study identified several factors related to developing severe COVID-19 infection in PLHIV. As in the general population, the severity of COVID-19 infection was found to be associated with either diabetes, hypertension, obesity, chronic kidney disease or cardiovascular disease [1, 11, 17]. This finding contributes to the understanding that comorbidities strongly correlate with severe COVID-19 outcomes [24–27].

Persistent immune dysfunction may be important in severe COVID-19 infection. This study indicated that a low CD4 count (less than 200 cells/mm^3^) was associated with COVID-19 severity. This finding was in accordance with other studies. Hoffman et al. showed that a current CD4 count of less than 250 cells/mm^3^ significantly correlates with the risk of severe COVID-19 [10]. In addition, Nomah et al. found that a low CD4 count (less than 200 cells/ mm^3^) was associated with worse outcomes from HIV-COVID-19 co-infection [11]. Jassat et al. showed that PLHIV with a history of CD4 count of less than 200 cells/mm^3^ were twice as likely to die in hospital than those with a CD4 count of 200 cells/mm^3^ [28]This may be due to the well-described lymphopenia that occurs in severe COVID-19 [29]. Zhang et al. evaluated studies reporting CD4 and CD8 count in severe COVID-19 patients, but not specifically PLHIV, indicating that both CD4 and CD8 T cell counts were significantly lower in the severe group compared to the non-severe group [30]. Therefore, both T cell counts may be considered as biomarkers for predicting severe COVID-19. Not using ART was also found to be a significant predicting factor of COVID-19 severity. HIV infection without ART can be a dangerous comorbidity of COVID-19 infection. Jassat et al. found that PLHIV who were not on ART were more likely to die in hospital than PLHIV who were on ART (aOR = 1.45, 95% CI 1.22–1.72) [28]. A study of T cell dynamics during COVID-19 infection has revealed that COVID-19 leads to a rapid augmentation of the T-cell exhaustion process initially caused by HIV, and this T cell degradation was observed to be the most pronounced in PLHIV not using ART [31].

To the authors’ knowledge, this is the first report on SARS-CoV-2 among PLHIV in Indonesia, and one of a limited number of studies that include PLHIV with a range of opportunistic infections. PLHIV with opportunistic infections are considered as part of the high-risk population for COVID-19 infection and the worse outcome population. In this study, most of the opportunistic infections diagnosed before or during COVID-19 infection were also severe infections, such as extrapulmonary tuberculosis, toxoplasma encephalitis, cryptococcal meningitis, histoplasmosis, and esophageal candidiasis. These kinds of infections also increase the risk of mortality among PLHIV. These findings also contribute important information about managing concurrent AIDS-defining illness during the COVID-19 pandemic. Though not statistically significant, a previous study in three European countries showed that 40% of its PLHIV population who had severe or critical COVID-19 infection had AIDS-defining illness while only 27% of PLHIV with mild to moderate COVID-19 had AIDS-defining illness [10]. The COVID-19 pandemic has disrupted public health priorities, including the fight against HIV. There have been some reports of late presenters of an AIDS-defining life-threatening condition as a result of difficulties accessing hospital care [32]. In addition, during the COVID-19 pandemic, there have been many reports indicating a reduction in HIV testing rates in many countries [33–35].

## Strength and limitations

This study has some limitations. First, the incidence of infection in the study population could not be precisely assessed because not all participants were tested for SARS-CoV-2. There may have been some symptomatic patients that did not get COVID-19 tested as the price of a SARS-CoV-2 PCR test in 2020–2021 was quite expensive, being almost one-fourth of the minimum wage during a critical epidemiological situation. The occurrence of asymptomatic COVID-19 patients can not be ruled out. However, these issues are reflected in the general population. Another limitation of this study arises from the retrospective design that did not allow for the evaluation of other factors that potentially influence the severity of COVID-19, such as smoking and HIV viral load.

Despite the limitations, a simple clinical scoring system that can predict severe COVID-19 infection among PLHIV was formulated. The 4-point scoring system covers the four risk factors included in the final multivariate model (ART status = 1, recent absolute CD4 = 1, opportunistic infection = 1, and any comorbidity = 1). This scoring system can predict COVID-19 severity outcome once PLHIV get infected with a good discrimination score, good calibration, and good internal validity. A total score of a minimum of 3 indicates a 58.4% probability of progressing to a COVID-19 severe for PLHIV once they have been diagnosed with COVID-19. While previous research focused on well-treated PLHIV, this study included a considerable number of PLHIV who were not on ART and had opportunistic infections, better depicting the situation in low-middle income countries. As both hospitalized and non-hospitalized COVID-19 patients were evaluated, this score can hopefully be used in outpatient and inpatient settings for PLHIV infected with SARS-CoV-2. Future studies will be needed to evaluate the performance of this score in different situations, especially for different SARS-CoV-2 variants and COVID-19 vaccine uptake among PLHIV.

## Conclusion

This study has identified four risk factors of severe COVID-19 infection in PLHIV, namely having any comorbidity, a recent absolute CD4 count of < 200 cells/mm^3^, not being on ART, and having an active opportunistic infection. This 4-point clinical scoring system has the potential to predict the COVID-19 severity progression of PLHIV diagnosed with COVID-19.

## Data Availability

The datasets used and/or analyzed during the current study is available from the corresponding author on reasonable request to the author’s email (evy.yunihastuti@gmail.com).

## Abbreviations

SARS-CoV-2: Severe acute respiratory syndrome coronavirus 2
COVID-19: Coronavirus disease 2019
ART: Antiretroviral therapy
SCOVHIV: Severity prediction score of COVID-19 in people living with HIV
PLHIV: People living with HIV
HIV: Human immunodeficiency virus
AUC: Area under the curve
PCR: Polymerase chain reaction
SD: Standard deviation
IQR: Interquartile range
CI: Confidence interval
AIDS: Acquired immunodeficiency syndrome
BMI: Body mass index
aRR: Adjusted risk ratios
SE: Standard error
HR: Hazard ratio
aOR: Adjusted odds ratio
